# Animal Models to Translate Phage Therapy to Human Medicine

**DOI:** 10.3390/ijms21103715

**Published:** 2020-05-25

**Authors:** Alessia Brix, Marco Cafora, Massimo Aureli, Anna Pistocchi

**Affiliations:** 1 Dipartimento di Biotecnologie Mediche e Medicina Traslazionale, Università degli Studi di Milano, LITA, Via Fratelli Cervi 93, Segrate, 20090 Milano, Italy; alessiam.brix@gmail.com (A.B.); marco.cafora@unimi.it (M.C.); massimo.aureli@unimi.it (M.A.); 2 Dipartimento di Scienze Cliniche e Comunità, Università degli Studi di Milano, Via San Barnaba 8, 20122 Milano, Italy

**Keywords:** phage therapy, bacteria, animal models, immune system, antibiotics

## Abstract

Phagotherapy, the use of bacteriophages to fight bacterial infections as an alternative to antibiotic treatments, has become of increasing interest in the last years. This is mainly due to the diffusion of multi-drug resistant (MDR) bacterial infections that constitute a serious issue for public health. Phage therapy is gaining favor due to its success in agriculture and veterinary treatments and its extensive utilization for human therapeutic protocols in the Eastern world. In the last decades, some clinical trials and compassionate treatments have also been performed in the Western world, indicating that phage therapy is getting closer to its introduction in standard therapy protocols. However, several questions concerning the use of phages in human therapeutic treatments are still present and need to be addressed. In this review, we illustrate the state of art of phage therapy and examine the role of animal models to translate these treatments to humans.

## 1. Phages for Therapy: Positive and Negative Outcomes

**Figure 1:**
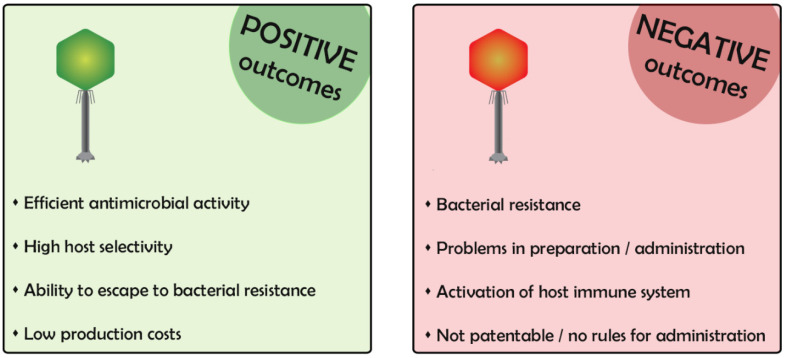
Positive and negative outcomes of phages.

Bacteriophages (phages) are viruses that specifically infect and multiply within the bacteria [1]. The use of phages to counteract bacterial infection dates to almost one century ago, and their use has never been abandoned completely, although it was mostly eclipsed by the advent of antibiotics. Nowadays, phages are regaining interest to overcome the development of antibiotic resistant bacteria [2]. However, phages are extremely appealing but also frightening in some aspects, particularly due to the incomplete knowledge of their mechanism of action. In the first part of this review, we describe phage characteristics, highlighting positive and negative aspects for their use in clinics (Figure 1).

The end point of phage infection is the death of the bacterium, usually through its lysis, and the release of progeny virions. Phages that have only this way of multiplication are called lytic phages and are suitable for phage therapy. Other phages, called lysogenic, may parasitize the host, leaving their genome inside the infected bacterium for generations [3]. In the lysogenic condition, the bacterium acquires immunity to superinfection of phages of the same type, a bad outcome for the purpose of phage therapy. Therefore, lysogenic phages are not used for therapy [4].

Another negative feature of several phages is their ability to transduce parts of the bacterial genome following the infection. This could cause the transmission of noxious or virulence genes in the bacterial population [5]. The possibility of the transducing ability of the phage used for therapy has to be checked. Moreover, some phages contain potentially harmful genes in their genomes. It is good practice to analyze the whole genome of the phage used for phage therapy to exclude it. Phage genome analyses could be a time-consuming technique incompatible with the need for urgent infection treatments in which phages are able to infect the bacteria of the patients and should be quickly identified and administered.

In phage therapy, it is appropriate to decide whether to use a single phage or a mix (cocktail) of phages with different characteristics. Given the high specificity of phage infection for a certain bacterial strain, the use of multiple phages is often better at containing an infection [6]. In particular, if the bacterial strain undergoes a mutation to resistance (e.g., mutation of the bacterial gene encoding the specific receptor necessary for phage adsorption or mutation in a bacterial function essential for phage reproduction), the presence of phages using different receptors or alternative functions will overcome the defeat of a single phage by the success of another. In addition, the use of phage cocktails that infect different bacterial species is often used in cases of skin infections in which different bacterial species are normally present [7].

Custom-designed phage cocktails are currently used for therapeutic treatments in countries of Eastern Europe, especially Georgia, where bacteriophages preparations are considered as pharmaceutics and prepared in authorized pharmacies. In Western countries, the situation is more complicated, mainly because no current rules for the use of phages as drugs have been developed until now. Indeed, as one of the main goals of phage therapy is the tailored treatment of an acute infection of an individual patient, it is difficult to apply the standard methods used for medical products (i.e., random and double-blinded clinical trials) for their commercialization. Moreover, non-engineered phages are natural compounds that cannot be patented, thus making their production of poor economic interest [8]. Recently, a few clinical trials have also been conducted in Western countries [9], and patient-tailored-phage therapy has been used for compassionate studies, such as to counteract *Acinetobacter baumanii*, *Pseudomonas aeruginosa,* and *Mycobacterium abscessus* infections [10,11,12]. Phage cocktails were prepared by combining the efforts of different laboratories and then intravenously injected in the patients and used in combination with antibiotics. All the published phage therapies were effective against the life-threatening disseminated infections of the patients.

Considering the time required for isolating phages from the environment, it would be of great interest to generate a phage bank containing libraries of characterized phages and a phage preparation storage at higher phage titer for rapid delivery, as is done in the Eastern countries [13]. One intriguing scenario could be the generation of a bank containing phages targeting all the multi-drug resistant (MDR) bacteria isolated from patients in each sanitary structure.

Phage preparation for human medical uses requires strict purification protocols to prevent endotoxin contamination. For studies in animal models, a sufficient degree of purification is achieved by CsCl gradient ultra-centrifugation [14] with subsequent endotoxin removal. Chromatographic methods can be also used for phage purification as well [15]. In chromatography-purified phages, endotoxin levels are decreased 10- to 30-fold with respect to the traditional method, but often the final phage titer is lower.

For human administration, the upper endotoxin (EU) threshold was defined at 5 EU/kg per h according to European Pharmacopeia regulations (FDA guideline, QAS11-452_FINAL_July12). Specialized institutes such as the Center for Phage Technology (CPT) or the Eliava Institute of Tblisi (Georgia) produce and provide large-scale, highly purified phages for clinical or research purposes [10,16,17,18,19,20]. Stability of phage preparations is essential to achieve efficient phage administration over time.

However, since each specific phage is different from another in its sensitivity to chemical and environmental factors, a universal strategy for their preparation is not possible yet. Usually, phages are resuspended in simple aqueous solutions. However, a gradual loss of phage activity can be observed during long-term storage of phage solutions, and, therefore, stabilizers must be added. Given the proteinaceous nature of phage capsids, protein stabilizers are usually added to phage preparations, including sugars (e.g., sucrose) and polymers (e.g., polyethylene glycol) [15]. Alternatively, phage solutions can be lyophilized and converted into powder with a high grade of stability [21].

## 2. Animal Models for Testing Phage Therapy

In the last years, several animal models of the most common and relevant human bacterial infections have been created and used to test newly isolated phages and their efficacy in fighting these pathogens in vivo [22]. Animal models of bacterial infection are necessary tools to (i) verify the efficacy of phage therapy in vivo, (ii) search for possible adverse effects, (iii) unravel interactions with the host (e.g., immune system activation). In the second part of this review, we describe how the generation of animal models of bacterial infections might help in the translation of phage therapy to human clinics.

### 2.1. Phage Therapy and Antimicrobial Action Using Invertebrate and Vertebrate Animal Models

**Figure 2:**
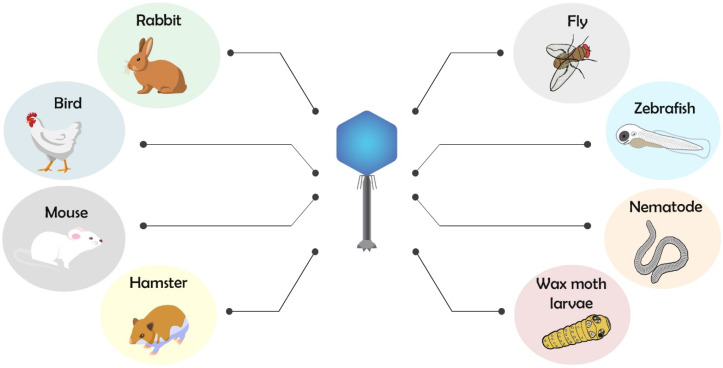
Animal models used for bacterial infection and phage therapy application.

Among the main used invertebrate or lower vertebrate models for phagotherapy, there are nematode (*Caenorhabditis elegans*), common fruit fly (*Drosophila melanogaster*), wax moth (*Galleria mellonella*), and zebrafish (*Danio rerio*), while for higher vertebrate, there are chicken (*Gallus gallus*), rabbit (*Oryctolagus cuniculus*), hamster (*Mesocricetus auratus*), and mouse (*Mus musculus*) models (Figure 2).

*C. elegans* is a small-size nematode (1 mm in length) that can be easily infected by bacteria, fungi, and virions inducing lethality of non-lethal infections [23,24]. The long list of pathogens infecting C. elegans also includes common human bacteria such as *P. aeruginosa*. Moreover, bacterial virulence factors that induce lethality in nematode are conserved in mammals, opening new opportunities in the use of *C. elegans* for large screening studies. While avoiding professional immune cells, in *C. elegans,* the defense to pathogens is mediated by epithelial cells that activate autophagy and the immune system though the production of antimicrobial proteins, peptides (AMPs), and p38 pathway activation [25]. The infection in nematodes can be easily achieved, as their nutritional source is the bacteria, thus pathogens primarily colonize the intestine, and phages can be delivered via the same route of administration. Augustine et al. (2014) and Glowacka-Rutkowska et al. (2019) [26,27] established *C. elegans* models for *Salmonella enteritidis* and *Staphylococcus aureus* infections and phage therapy application. In both cases, the bacteriophage administration resulted in a considerable increase in the survival of infected larvae. Remarkably, the healthy state of the recovered nematodes was confirmed by the fact that they produced healthy progeny after 100 h after phage treatment. Although these two studies take into account the mortality as a unique parameter for testing a phage’s efficacy and effects, the results indicated that *C. elegans* can be a useful animal model for these studies.

Among non-vertebrate infection models, insects have a strong potential due to their complex innate immune system, which shows high similarity to those of mammals [28,29]. Moreover, they are considered suitable alternative models to larger mammals for bacterial colonization studies and excellent tools for pharmacokinetic studies of antimicrobials [28,30,31]. In two different studies, *D. melanogaster* was used to evaluate the therapeutic effect of phages against *P. aeruginosa* infections. In the first study done by Lindberg et al. (2014) [32], the authors investigated the pharmacokinetics and the possible toxicity of phages by themselves. Phage solutions were mixed to corn meal-dextrose medium and administered to healthy flies. The presence of live bacteriophages in the flys’ lysates at different time points after treatment demonstrated that phages survived and were not degraded in the gastrointestinal system. This suggests that oral administration can be successfully studied in animal models, highlighting the interesting possibility of using *D. melanogaster* to test oral administration of phages. Moreover, the absence of lethality after phage administration indicates that phage treatments are safe and free of toxicity. In the second work done by Heo et al. (2009) [33], the authors compared the effects of *P. aeruginosa* infection and phage administration in mice and D. melanogaster. The use of two infection models is important to confirm the antibacterial activity of phages against *P. aeruginosa* that activates different virulence factors depending on the host. Given the promising potential of *D. melanogaster* as a simple, rapid and cheap animal model to conduct studies on bacterial infection and phage therapy, a guided protocol has recently been set up to evaluate the antibacterial efficacy of new bacteriophages against *P. aeruginosa* infection in this model [34].

Another invertebrate used for microbial infection and phage therapy is *G. mellonella*. In a study done by Seed et al. (2009) [35], different bacteriophages were efficiently administrated in *G. mellonella* larvae to treat *Burkholderia cepacia* infection. The authors also addressed if the protective effect observed in the treated larvae was due to bacteriophages’ action rather than to the reaction of the immune system of the host triggered by the phage injection. They found that heat-inactivated phages activated the immune system but did not improve larvae survival, indicating that the antibacterial action depended on active phage multiplication. Interestingly, two different studies in wax moth larvae also reported a prophylactic efficacy when phage cocktail was injected or orally administrated. In the first study done by Nale et al. (2016) [36], a four-phage cocktail able to disrupt *C. difficile* biofilm was effective in increasing survival when added to the larvae food, preventing bacterial colonization. In a second study done by Forti et al. (2018) [37], the six-phage cocktail initially used to prevent *P. aeruginosa* infections in *G. mellonella* efficiently counteracted lung infections in the mouse. This result showed that the same bacteriophages can function in different species, both invertebrates and vertebrates.

Zebrafish is gaining favor as a model for the study of host-bacterial interactions, especially in its embryonic stage [38,39,40]. The presence of a developed innate immune system, genetic tractability, and optical transparency of the embryos make it useful for studying aspects of infectious diseases not accessible in traditional animal models. Recently, some zebrafish models were set up to study bacterial infections such as *Enterococcus faecalis* and *P. aeruginosa* for phage therapy application [41,42]. Systemic infection in zebrafish embryos is performed through the injection of bacteria in the circulation followed by phage administration via the same route. The success of phage therapy treatment was demonstrated by an increased survival of the infected zebrafish embryos, their recovery from the altered morphology caused by bacterial infection, and decreased bacterial burden after plating homogenized embryos. This vertebrate model validates the efficiency of phage therapy in a quick (five days) and cheap way and demonstrates the survival and the efficacy of phages delivered in the blood with an aquatic model.

The use of invertebrates and lower vertebrates such as zebrafish presents several advantages for the research, such as reduced cost and experimental procedure time. However, to translate phage therapy to humans, it is also necessary to use higher vertebrate models. For instance, in birds, oral phage administration was applied as prophylaxis or post-infection treatment to counteract salmonellosis, colibacillosis, and campylobacteriosis infections that represent a worldwide economic and health problem in poultry [43,44]. Some studies also investigated the use of encapsulated phages of part of the virion, such as the tailspike domain, to improve phage therapy in chickens [45,46,47]. Given the importance of phage therapy application using birds as animal models, a procedure to test phage efficacy using a chicken embryo of colibacillosis infection was recently described [48].

Rabbits were also used as a model of *S. aureus* wound infection followed by phages administration [49]. As with humans, but contrary to mice, rabbits naturally suffer for *S. aureus* infections, representing a suitable animal model to study the invasion of these pathogens. The bacterial infection was performed by subcutaneous injections that generated abscesses. Phage administration was performed simultaneously to bacteria or immediately after but in the same location. To evaluate the efficacy of phage therapy, animals were killed four or six days after infection, and the bacterial load in the abscesses area was evaluated. In another study presented by Kishor et al. (2016) [50] and commented upon by Abedon (2016) [51], phage therapy was tested in a rabbit model of *S. aureus* infection. Although authors demonstrated the feasibility of phage therapy to cure bacterial infection, the rabbit model was different from the patient’s situation in which bacterial infection was chronic, and phage therapy was applied after the failure of conventional approaches. A prophylactic effect of phages to prevent or reduce bacterial infection was demonstrated in new-born mouse and rabbit models infected by *Vibrio cholerae* by Yen et al. (2017) [52].

An interesting work by Nale et al. (2016) [53] demonstrated that hamsters infected with *Clostridium difficile* and orally administered with phage cocktail showed increased survival rate. Due to the lack of virulent lytic phages infecting *C. difficile*, in this study, temperate phages were used. Thus, it was unsuitable for therapeutic treatment. However, the authors showed how the combination of multiple phage types might limit their harmful impact.

Among mammals, murine models are the most frequently used to study phage therapy. Given their high similarity with humans, they have been used not only to demonstrate the efficacy of the classical phage therapy [37,54,55,56,57] but also to investigate the interactions between phages and the host immune system. This second issue is treated in detail in the following chapter.

In Table 1, we resume the studies on animal models and antibacterial activity of phages.

### 2.2. Phages and Immune System Interactions Studies Using Animal Models

Mouse models of bacterial infection and phage treatment have also been used to investigate different aspects of phage activity in vivo, such as the interaction between phages, bacteria, and the host immune system. For example, Abd El-Aziz and colleagues (2019) [63] used a mouse model of *P. aeruginosa* infection to investigate the synergism between phages and the innate immunity of the host considering the activity of phages in serum. When the serum and the phages were added to the bacterial culture, an increased antimicrobial activity of phages was achieved. On the contrary, when heat-inactivated serum was added, the phage antimicrobial activity was not increased. This might explain why phages are more efficient in counteracting bacterial infection when administered intravenously than in lungs of infected mice.

Roach et al. (2017) [64] used immunodeficient mice *Myd88−/−, Rag−/−, Il2rg−/−*, and a neutrophil-depleted line to dissect the contribution of immunity cells to phage–host synergy. Upon *P. aeruginosa* infection, only the neutrophils-depleted mice were completely unresponsive to phage treatment. *Rag−/− and Il2rg−/−* mice lacking two key genes for lymphocyte function behaved as the wild-type. An intermediate situation with an initial response followed by the proliferation of phage-resistant bacteria was achieved when Myd88, the main adaptor for Toll-like Receptor (TLR) pathway, was depleted. Both studies suggest an interplay between phages and innate immune response involving complement cascade pathway and neutrophils activity—an important aspect to consider in view of phage therapy application to immunodeficient patients. Another study done by Trigo et al. (2013) [60] investigated the immune response in a mouse footpad infected by *Mycobacterium ulcerans*. Subcutaneous phage administration reduced bacterial proliferation both in the skin and in the draining lymph nodes, thus preventing ulcerations. Moreover, histopathological analyses of the necrotic tissues of phage-treated mice showed extended macrophages and lymphocytic infiltrates, which colocalize with few residual bacteria, indicating a phage-mediated activation of phagocytes and adaptative response.

Immune response and phage therapy were addressed also by Cafora et al. (2019) [42] in a zebrafish model of cystic fibrosis (CF). A *P. aeruginosa* systemic infection was generated in CF zebrafish embryos and treated effectively by phage administration. In addition, a decrease of pro-inflammatory cytokine levels was observed in phage-treated infected embryos. Interestingly, CF zebrafish embryos showed a basal inflammatory status in the absence of bacterial infection similar to the high inflammation presented in human CF patients. Phage administration in CF embryos reduced the levels of pro-inflammatory cytokines, suggesting that phages might modulate the innate immune system and therefore opening the possibility to use bacteriophages as anti-inflammatory agents in conditions of constitutive inflammation.

Another important aspect is phage immunogenicity, which is the aptitude of phages to induce specific immune responses with the production of specific antibodies against phage antigens. Opinions and data collected about phage immunogenicity in humans are few and contradictory. Importantly, some clinical outcomes indicate that phages widely vary in their immunogenicity depending on phage-type, dose, route of administration, and host immune status. In general, no strict dependence between phage treatment efficacy and level of antiphage-antibodies emerged [65,66,67,68]. Animal models contributed to shed light on this debatable question. The dynamics of phage immunogenicity was studied by Capparelli et al. (2007) [61] in a mouse model in which a dose of 10^7^ plaque forming units (pfu) of *S. aureus* phages were intravenously administrated at intervals of two weeks. Phage presence persisted in the blood circulation for approximately 21–25 days and, although antibodies against phages were present, they did not neutralize phage-antibacterial activity. Similar immunogenicity tolerance was obtained by Roach et al. (2017) [64] in a mouse model intranasally inoculated with a single dose of approximately 10^9^ pfu of *P. aeruginosa* phages; a high degree of phage persistence was observed in the airways of all tested mice in the four days after the administration. Another study in mice was performed by Majewska et al. (2015) [69] in the absence of bacterial infection to analyze the immunological response of long-term exposure of T4 phage (100 days of continuous administration). The T4 phage was administrated in the drinkable water at a concentration of 4 × 10^9^ pfu/mL, and the evaluation of Immunoglobulin (Ig)M, IgG, and secretory IgA production in serum and gut was correlated to the microbiological profile of the mouse at day 240. IgM induction was detected only after IgG boost that started from 36 days and remained sustained even after phage removal from the diet. IgA secretion was detected after 79 days and gradually decreased after T4 removal. After 100 days, the production of IgG was dependent on the phage titer administration (higher dose 4 × 10^9^ pfu/mL, lower dose 4 × 10^8^ pfu/mL). These studies using animal models suggest that specific phage-humoral responses could occur, but it might be dependent on route, dose, and time of administration.

An interesting point concerns the T-cell proliferative rate in response to phages. In a study done by Kim et al. (2008) [70], explanted murine T-cells exposed to salmonella phages showed a higher proliferative response when the donor mice were pre-exposed in vivo to salmonella phages in comparison to T-cells isolated from non-pre-treated mice, suggesting that phages might activate per se the host immune system. Phage proteins differ in their capacity to activate humoral responses. Indeed, Dabrowska et al. (2014) [71] showed that a pre-immunization of mice with purified T4 phage capsid proteins gp23, gp24, Hoc, or Soc impaired the antimicrobial activity of T4 when mice were infected with *Escherichia coli* and administered with the phages. These important results obtained in vivo in an animal model of infection demonstrated that phages may have reduced activity to counteract bacteria as they are subjected to specific immunization due to phage capsid proteins.

### 2.3. Route of Phage Administration in Animal Models

Phagotherapy is efficient only when a sufficient amount of phages are able to reach the bacteria and kill them [72]. Three main routes of phage administration have been tested in human clinical trials or case reports depending on the infection type and the localization: topical, intravenous, and oral. Each of these routes presents some complications that still must be solved before translation of phage therapy in human standard medical procedures. Animal models represent a useful tool to investigate these aspects and to optimize phage application in humans.

Topical phage application has been largely used for the treatment of bacterial infections associated with ulcers, surgical wounds, or burns [73]. Recently, several cases of compassionate bacteriophage treatments of diabetic foot ulcers have been reported as successful [18,19]. This is of particular importance, as diabetic patients are commonly immunocompromised with nephropathy and hepatic insufficiency, and prolonged antibiotic treatments are not well tolerated. No animal models of diabetic ulcers are available to test phage therapy to date, but several mouse models of skin ulcers, burn wounds, and infections were topically treated with phages [59,60,74] safely and without adverse effects. These data in mice suggest that ineffective results in two small human burn trials [75,76] are probably linked to an incorrect combination of modality, doses, and times of application rather than to ineffectiveness of phage therapy.

Besides treatment of epithelial lesions, topical phage therapy has been successfully used to treat infections of specific tissues or organs. For example, inhalation of phage solutions has proven to be effective in counteracting MDR bacterial lung infections both in humans [77] and in mouse models [62,78].

Although the size and the immune systems of animal models are different from humans, a pre-screening of phage compound in animals might at least optimize treatment schedules and anticipate any adverse/toxic effects possibly elicited by topical phage administration.

Intravenous (IV) administration consists of injecting the phage preparations directly in the bloodstream, an ideal route for the treatment of bacteremia or widespread infections. The study of Speck and Smithyman (2015) [79] presented IV phage therapy as safe and effective, but some aspects have been questioned. One of the most common objections is that the phage’s lytic activity could lead to the release of great amounts of endotoxins directly in the bloodstream, triggering a strong immune response and anaphylaxis. Notably, Duplessis et al. (2008) [80] reported an anaphylactic response in a pediatric case after IV phage administration, and the authors did not exclude a link to endotoxin release.

Moreover, phages could be rapidly cleared from the blood, losing their effectiveness. These concerns must be deeply investigated prior to introducing IV phage therapy in standard medical procedures. Therefore, several studies about the pharmacokinetics of phages administered with IV injection have been conducted in rodents. Dąbrowska (2016) [81] demonstrated that, after IV injection, phages are not simply diluted in the body volume but are probably neutralized by anti-phage antibodies and removed from the bloodstream by the phagocyte system.

Oral administration of bacteriophages involves gastro-intestinal transit and might have implications for the entire organism. Two main issues must be considered: first, endotoxins released by lysed bacteria can be absorbed and enter the blood stream, leading to systemic inflammation; second, the endogenous microbiome, which is crucial for digestive processes and defense against pathogens, can be altered.

Several safety tests have been done to verify the efficacy of oral administration and exclude possible adverse effects due to the presence of the phage itself [16,68,82]. These studies also reported that the overall composition of the microbiota remains quite stable upon phage administration. Although active phages were found in fecal samples after oral administration, it is not clear if phages can bypass the gastric environment without being killed massively by the acid solutions. Again, in vivo studies using *Drosophila* as an animal model provide important evidence that phages can be orally administered with food and survive through the gastro-intestinal tract [33]. Another study on pigs done by Jamalludeen and colleagues (2009) [58] showed that an anti-acid pre-treatment with sodium bicarbonate significantly increases the number of active phages in feces and reduces the severity of diarrheal symptoms. Alternatively, alginate/CaCO_3_ microencapsulation decreases phage degradation in the gastric environment, thus ameliorating phage efficacy as reported by Colom and colleagues using a chicken model of *S. enterica* infection [45].

### 2.4. Methods to Improve Phage Therapy Using Animal Models

One of the most promising aspects of phage therapy is the possibility to perform combined treatments with antibiotics commonly used to treat bacterial infection. This is of particular interest, as it reduces the time and the doses of antibiotic administration, diminishing the development of antibiotic-resistant bacteria and adverse effects of the drugs on the host (i.e., microbiome destruction). A successful combined treatment of phages and antibiotics was described by Cafora et al. (2019) [42] in the zebrafish CF model with *P. aeruginosa* infection. The reduction in mortality rate following phage cocktail administration was greater when phages were combined with ciprofloxacin. Moreover, when bacteria mutate and become phage resistant, it is possible to isolate new phages using the phage-resistant mutants. Although it is time consuming, this method could be used to overcome resistance in bacteria. Another important consideration is that the bacteria that become phage-resistant display increased sensitivity to the antibiotic treatment that previously failed [83]. For instance, Schooley et al. (2017) [10] showed that a minocycline isolate of *Acinetobacter baumannii* from a patient with severe pancreatitis displayed reduced minocycline resistance after phage therapy.

Phages may also be considered for prophylaxis to prevent or reduce infections of different bacterial species, as demonstrated in several animal models [36,37,43,54,56,58].

A further interesting possibility to improve phage therapy is the use of liposomes and transfersomes for phage delivery [84]. These vesicles act by creating a broader distribution, preventing rapid degradation and enhancing cellular uptake [85,86]. This possibility was explored in a study done by Chhibber and colleagues (2017) [87] in a rat model of acute skin and soft tissue infection of *S. aureus* in which transfersomes-entrapped phages performed a faster rescue than free phages. A similar effect of improved phage efficacy was achieved in Colom et al. [46] by using liposome-entrapped phages in a chicken model of *S. enterica* infection. Therefore, liposomes and transfersomes could be a useful tool to potentiate the efficacy of phage activity.

Last but not least, many studies successfully demonstrated the antibacterial action of some phage enzymes, such as endolysins, virion-associated lysins, and capsular depolymerases [88]. Several studies about phage-derived lysins have been concluded both in animal models and in humans [89], and a recent clinical trial demonstrated the safety of endolysin intravenous administration [90]. In contrast to lysins, depolymerases do not lyse bacterial cells, thereby preventing endotoxin release and inflammation. Moreover, after capsule removal by depolymerases, bacteria are directly exposed to the host immune system and can be more easily killed and removed. The efficacy of depolymerase treatment was demonstrated in vivo in a mouse model of *E. coli* infection [91]. The same treatment successfully resulted in the rescue of both immunocompetent and leukopenic mice, with a higher efficiency when the enzyme was administrated shortly after infection [92]. One major limitation in the use of depolymerases is that, while phages can replicate autonomously in bacterial cells, enzymes cannot, and therefore the administration of a single dose could not resolve the infections.

## 3. Conclusions

In conclusion, phages proved time and again to have the appropriate characteristics to be introduced in human clinical treatments. Although several concerns are still present in Western countries, a progressive improvement of their use in clinical trials or for compassionate studies has been achieved. Before translating phage therapy to human clinics, some points still need to be clarified and excluded, as described in this review.

Animal models are an important tool to further understand the mechanisms and the efficacies of bacteriophage action in vivo. Both invertebrates and vertebrates demonstrate the success of such treatments in a way that is cheaper, faster, and more ethical than human clinical trials. The generation of vertebrate animal models to test phages may give a more comprehensive understanding of the mechanisms triggering host immune and inflammatory response to phages, one of the most important concerns of phage therapy application to humans. Several strategies to improve and potentiate phage activity have been tested, underlining the promising strength of bacteriophages or their enzymes in human therapies. Among these, we cited the possibility to improve antibacterial action by enhancing phage delivery, the use of phages mixed in cocktails, the combination of phages with antibiotics, and the possibility to use phages for prophylactic treatments. All these studies can be easily (and cheaply) achieved in animal models before translation to humans.

Moreover, considering that one of the most important goals of modern phage therapy is to rapidly identify phages able to counteract bacterial infection in compassionate studies, animals can be used to indicate the safety of select phages before patient treatment. Since the custom-made use of phages cannot be regulated right now, phage pre-screening in animals for personalized therapies could at least limit some of the concerns [22].

For all these reasons, animal models are a key tool in investigating potential phage therapies and introducing them to human medicine.

## Figures and Tables

**Table 1:** Animal models of human phage therapy for common human pathogens.

Animal Model	Challenge (Pathogen)	Condition	Phage Treatment	Route of Administration	Results Summary	Reference
*C. elegans*	*Salmonella enterica*; spread on agar plate	lethal systemic infection	mono-phage, delay (24 hpi); 5 × 10^9^–1× 10^10^ pfu	in growth medium	>survival rate	Augustine et al., 2014 [26]
*C. elegans*	*Staphylococcus aureus*; spread on agar plate	lethal systemic infection	mono-phage, delay (24 hpi); 10^9^ pfu/ml	in growth medium	>survival rate	Glowacka-Rutkowska et al., 2019 [27]
*D. melanogaster*	*Pseudomonas aeruginosa*; intrathorax injection of 10^3^ cfu/fly	lethal systemic infection	mono-phage, delay (6 hpi); 10^4^ pfu/fly	intrathorax injection	>survival rate	Lindberg et al., 2014 [32]
*D. melanogaster*	*Pseudomonas aeruginosa*; intrathorax injection of 50–200 cfu/fly	lethal systemic infection	mono-phage, co-adm; 10^6^ pfu/fly	oral (force feed)	>survival rate; <BB	Heo et al., 2009 [33]
*G. mellonella*	*Clostridium difficile*; oral administration 10^5^ cfu/larva	lethal systemic infection	4-phage cocktail: proph (2 hbi), delay (2 hpi) or co-adm; 1 to 4 doses of 10^6^ pfu/larva	oral	reduced mortality (100% in proph); dose-dependence	Nale et al., 2016 [36]
*G. mellonella*	*Burkholderia cepacia*; injection of 2.5 × 10^3^ cfu/larva	lethal bacteremia	mono-phage, delay (6 or 12 hpi); 2.5 × 10^3^ pfu/larva	injection	>survival rate; <BB	Seed et al., 2009 [35]
*G. mellonella*	*Pseudomonas aeruginosa* (lab and clinical strains); injection of 30 cfu/larva	lethal bacteremia	6-phage cocktail: proph (1 hbi) or delay (1 hpi); 1.5 to 4.5 × 10^3^ pfu/larva	injection	prolonged survival time after infection	Forti et al., 2018 [37]
*G. mellonella*	*Acinetobacter baumanii* (XDR); injection of 5 × 10^5^ cfu/larva	lethal bacteremia	2-phage cocktail or mono-phage, delay (0.5 hpi); 5 × 10^7^ pfu/larva	injection	>survival rate (≥80%)	Leshkasheli et al., 2019 [57]
Zebrafish	*Enterococcus faecalis* (clinical strain); injection in circulation of 3 × 10^4^ cfu/embryo	lethal systemic infection	mono-phage, delay (2 hpi); 6 × 10^5^ pfu/embryo in 2 nL	injection in circulation	>survival rate (of 57%); >healthy state	Al-Zubidi et al., 2019 [41]
Zebrafish	*Pseudomonas aeruginosa*; injection in circulation of 30 cfu/embryo	lethal systemic infection	4-phage cocktail, delay (0.5 or 7 hpi); 500–1000 pfu/embryo in 2 nL	injection in circulation	>survival rate (of about 30%); <BB; reduced inflammatory response	Cafora et al., 2019 [42]
Quail	*Salmonella enterica* (Enteriditis); oral administration of 1.2 × 10^8^ cfu/quail	gastrointestinal infection	mono-phage, proph or delay *; 10^5^ pfu/mL, 3 doses daily	oral (oral gavage or vent lip)	<BB in cecal tonsils	Ahmadi et al., 2016 [43]
Chicken	*Salmonella enterica* (Typhimurium); oral administration of 10^7^ cfu/chicken	gastrointestinal infection	3-phage cocktail (liposome/alginate encapsulated), delay (24 hpi); 10^9^/10^10^ pfu/chicken, 8 doses daily	oral	<BB in cecum (of 1.5–3.9 Log_10_ cfu)	Colom et al., 2015, 2017 [45,46]
Rabbit	*Staphylococcus aureus*; subcutaneous injection of 8 × 10^7^ cfu/rabbit	local infection (abscess)	mono-phage, co-adm or delay (6, 12 or 24 hpi); 2 × 10^9^ pfu/rabbit	subcutaneous injection	<BB of infected area and abscesses prevention in co-adm (no effect in delay)	Wills et al., 2005 [49]
Rabbit	*Staphylococcus aureus* (MRSA); Intramedullary injection of ≤5 × 10^6^ cfu/rabbit (*)	chronic osteomyelitis	7-phage cocktail, delay (21, or 42 dpi); 5 × 10^11^ pfu/rabbit, 4 doses total every 2 days	Intralesional injection	cure of infection in 21 dpf treatment	Kishor et al, 2016 [50]
Rabbit	*Vibrio cholerae*; oral administration of 5 × 10^8^ cfu/rabbit	gastrointestinal infection	3-phage cocktail: proph (3 or 24 hbi); 4–8 × 10^9^ pfu/rabbit	oral	prevention of diarrheal symptoms; <BB in intestine (of 1–4 Log_10_ cfu)	Yen et al., 2017 [52]
Hamster	*Clostridium difficile*; oral administration of 2 × 10^3^ spores/hamster	gastrointestinal infection	2,3,4-phage cocktails or mono-phage, delay *; 8 × 10^7^ pfu/mL, every 8 h × 36 hpi	oral	<BB in cecum and colon (of 2 Log_10_ cfu)	Nale et al., 2017 [53]
Pig	*Escherichia coli* (ETEC); oral administration of 10^10^ cfu/pig	gastrointestinal infection	2,3-phage cocktail or mono-phage, proph (0.25 hbi, 3 × 10^9^–10^10^ pfu/pig) or delay (24 hpi, 6 doses every 3 h, 10^8^ pfu/pig)	oral	diarrhea symptoms ameliorate	Jamalludeen et al., 2009 [58]
Murine	*Pseudomonas aeruginosa*, intranasal injection of 1 × 10^7^ cfu/mouse	lethal respiratory infection	6-phage cocktail, delay (2 hpi); 10^7^ pfu/mouse	intranasal injection	100% reduced mortality; <BB (about 3 Log_10_ times)	Forti et al., 2018 [37]
Murine	*Pseudomonas aeruginosa*, intranasal injection of 2.5 × 10^7^ cfu/mouse	respiratory infection	3-phage cocktail: proph (48 hbi), co-adm or delay (24 hpi); 1.24 × 10^9^ pfu/mouse	intranasal injection	>survival rate; bacterial clearance in BALs (proph 71%, co-adm 100% and delay 86%); reduced inflammatory response	Pabary et al., 2016 [54]
Murine	*Pseudomonas aeruginosa* (MDR), intraperitoneal injection of 10^7^ cfu/mouse	lethal bacteremia	mono-phage, co-adm; 1 ×10^9^ pfu/mouse	intraperitoneal injection	85% reduced mortality; bacterial clearance in blood; reduced inflammatory response	Alvi et al., 2020 [55]
Murine	*Pseudomonas aeruginosa* (clinical strain), intranasal injection of 10^7^ cfu/mouse	lethal lung infection	mono-phage, proph (24 hbi) or delay (2, 4, 6 hpi); 10^8^ pfu/mouse	intranasal injection	>survival rate: delay-dependent (from 100% in 2 hpi to 25% in 6 hpi) and 100% in proph; reduced inflammatory response	Debarbieux et al., 2010 [56]
Murine	*Acinetobacter baumanii* (XDR), intraperitoneal injection of 6 × 10^7^ cfu/mouse	lethal bacteremia	2-phage cocktail or mono-phage, delay (2 hpi); 6 × 10^9^ pfu/mouse	intraperitoneal injection	>survival rate (≥80%)	Leshkasheli et al., 2019 [57]
Murine	*Klebsiella pneumoniae*, topical administration 50 ul of 10^8^ cfu/mL	burn wound infection	5-phage cocktail or mono-phage, delay (6 hpi); 50 uL of 10^8^ pfu/ml	topical	<BB in skin tissue; faster wound healing; reduced inflammatory response	Chadha et al., 2016 [59]
Murine	*Mycobacterium ulcerans*, subcutaneous injection of 10^5.5^ afb	local infection (ulceration)	mono-phage, delay (33 dpi); 10^8^ pfu/mouse	subcutaneous injection	<BB in skin tissue; prevent ulceration	Trigo et al., 2013 [60]
Murine	*Staphylococcus aureus* (MRSA), subcutaneous injection of 10^7^ cfu/mouse	local infection (abscess)	mono-phage, co-adm or delay (4 dpi); 10^9^ pfu/mouse	subcutaneous injection	prevent/ameliorate abscess development	Capparelli et al., 2007 [61]
Murine	*Staphylococcus aureus* (MRSA), intravenous injection of 10^8^ cfu/mouse	systemic infection	mono-phage, co-adm; 10^9^ pfu/mouse	intravenous injection	97% reduced mortality; bacterial clearance in blood	Capparelli et al., 2007 [61]
Murine	*Klebsiella pneumoniae*, intranasal instillation of 10^9^ cfu/mL	Lung infection	mono-phage, delay (2 hpi); 10^9^ pfu/mouse	intranasal instillation	<BB in lung and serum; prevent severe lung lesions	Anand et al., 2019 [62]

hbi = hours before initial infection; hpi = hours post initial infection; dpi = days post initial infection; cfu = colony-forming-units; pfu = plaque-forming-units; afb = acid fast bacilli; BB = bacterial burden (or bacterial load); delay = delayed treatment; co-adm = co-administration; proph = prophylactic treatment; MDR = multi drug resistant; XDR = extensively drug resistant; MRSA = methicillin-resistant *Staphylococcus aureus*; ETEC = enterotoxigenic *E. coli*; BAL = bronchoalveolar lavage; n° of doses = 1 if not differently indicated; * = not described.

## Abbreviations

MDR	Multi drug resistant
CF	Cystic fibrosis
EU	Endotoxin units
FDA	Food and drug administration
CPT	Center for Phage Technology
IV	Intravenous
PFU	Plaque forming units
